# Physician admission preferences under the Diagnosis-Intervention Packet payment system: a discrete choice experiment in a Chinese tertiary hospital

**DOI:** 10.3389/fpubh.2026.1946819

**Published:** 2026-10-22

**Authors:** Yuanming Song, Siyue Zheng, Mingkai Zhao, Zunzun Nie, Shuaihe Zhu, Kang Li, Xuefeng Duan, Yuxue Feng

**Affiliations:** 1 First Affiliated Hospital, Henan Medical University, Xinxiang, China; 2 Faculty of Education, Henan Normal University, Xinxiang, China; 3 School of Medical Humanities, Henan Medical University, Xinxiang, China

**Keywords:** Diagnosis-Intervention Packet (DIP), discrete choice experiment, health economics, payment system, physician preferences, provider incentives

## Abstract

**Background:**

Payment reforms seek to contain costs while maintaining quality, but their effects depend on how frontline physicians respond to financial and regulatory signals. China’s Diagnosis-Intervention Packet (DIP) reform is a case-based, point-based payment model implemented within a regional global budget.

**Objective:**

To examine physicians’ admission preferences and trade-offs across clinical, financial, operational, and regulatory attributes within a tertiary hospital operating under the DIP payment system.

**Methods:**

We conducted a cross-sectional discrete choice experiment among 339 physicians in a tertiary hospital in central China. Six attributes captured clinical complexity, length of stay, treatment technology, service revenue, expected DIP margin, and compliance risk. Physicians’ choice preferences and opt-out choices were analyzed using mixed logit models, and preference heterogeneity was further examined using latent class logit models.

**Results:**

Expected DIP loss was strongly negatively associated with admission preference (*β* = −1.81, *p* < 0.001), whereas expected profit was associated with a smaller positive preference (*β* = 0.45, *p* < 0.001). A Wald test confirmed a significantly stronger response to losses than gains (*p* < 0.001), consistent with loss aversion. Expected DIP margin accounted for 41.94% of relative attribute importance, followed by clinical complexity (20.11%) and compliance risk (16.01%). Opt-out analysis showed that expected loss, higher clinical complexity, above-benchmark length of stay, and compliance risk were associated with a greater tendency to select the opt-out option. Five distinct preference profiles were identified.

**Conclusion:**

Physician admission preferences under DIP were associated with financial, clinical, operational, and regulatory factors, with substantial heterogeneity across physicians. These findings may inform coordinated refinements in payment design, regulatory oversight, and hospital management.

## 1 Introduction

Healthcare systems worldwide face the dual challenge of expanding access to care while maintaining fiscal sustainability. Globally, approximately 2.1 billion people experience financial hardship in accessing healthcare, and 1.6 billion are pushed into or further into poverty due to medical expenses (1). At the same time, growth in government health expenditure is slowing, particularly in low-and middle-income countries (2). China illustrates this policy tension. In 2024, the growth rate of revenue for the employee medical insurance fund (3.5%) was outpaced by expenditure growth (7.6%), indicating sustained fiscal pressure (3). Because China operates the world’s largest basic medical insurance system, its provider payment reforms have both domestic relevance and broader implications for countries seeking to align cost containment with care quality.

Against this background, China has moved from fee-for-service toward a multipayer, composite payment system (4). The earlier fee-for-service model lacked effective cost containment and often encouraged overprovision of drugs and tests (5). To strengthen provider incentives, the National Healthcare Security Administration piloted Diagnosis-Related Groups (DRG) in 30 cities and Diagnosis-Intervention Packet (DIP) payment in 71 cities between 2019 and 2021. The Three-Year Action Plan for DRG/DIP Payment Reform set the goal of nationwide coverage by 2025 (6). DIP is a case-based, point-based payment reform implemented within a regional global budget. Cases are categorized according to diagnosis-procedure combinations, assigned points, and settled according to accumulated points and local point values (7). Compared with DRG, DIP places lower requirements on medical record standardization and health information system maturity, which may facilitate implementation in less developed regions (8).

Most evaluations of payment reform examine system-level outcomes, such as case costs, length of stay, resource use, or fund expenditure (9–12). Such studies are indispensable, but they do not explain how the policy signals embedded in payment rules are translated into frontline clinical decisions. This distinction matters because unintended responses to payment reform, including cost-shifting, upcoding, and possible underprovision, ultimately arise from the way providers adapt to economic and regulatory incentives (13–16). Traditional retrospective data are limited in their ability to isolate how physicians trade off clinical severity, expected financial margin, regulatory exposure, and service value when making admission decisions.

This study uses a discrete choice experiment (DCE) to characterize physicians’ stated admission preferences and trade-offs within the DIP payment context. Grounded in random utility theory and Lancaster’s characteristics theory, DCEs quantify individual trade-offs across attributes by asking respondents to choose among hypothetical alternatives with systematically varied levels (17–20). The DIP setting provides a relevant context for this approach because admission choices may involve simultaneous clinical, financial, operational, and compliance considerations. Accordingly, we used mixed logit and latent class models to examine physicians’ admission preferences and preference heterogeneity. Specifically, this study addressed three interrelated questions: how physicians trade off clinical, financial, operational, and regulatory attributes when making admission choices; whether these attributes are associated with physicians’ willingness to admit patients in hypothetical choice scenarios; and whether admission preferences exhibit heterogeneity across physicians. Together, these analyses aimed to characterize physicians’ preference structures within the DIP payment context and provide evidence relevant to payment design and hospital management.

## 2 Materials and methods

### 2.1 Study design

This study employed a discrete choice experiment (DCE), with policy document review and semi-structured interviews used to inform the experimental design. First, 16 key national policy documents relevant to medical insurance payment reform and the development of DIP, issued by relevant Chinese central government authorities between 2016 and 2025, were systematically reviewed to identify the main policy objectives and incentive mechanisms (Supplementary material). Subsequently, semi-structured interviews were conducted with eight key informants, including three senior clinicians from pediatrics, urology, and oncology, and five hospital administrators responsible for medical insurance, medical records, quality management, medical affairs, and hospital operations, respectively. Findings from the policy document review and interviews were jointly used to develop and refine the DCE attributes, levels, and choice scenarios. Physicians’ preference data were ultimately elicited through simulated patient admission decisions.

### 2.2 Study participants and sampling

Participants were clinical physicians from a tertiary hospital in Henan Province, China. The hospital has implemented DIP payment for all inpatient cases since 2023, covering more than 3,000 DIP groups. To ensure representation across major clinical specialties, physicians were recruited from internal medicine, surgery, obstetrics and gynecology, and pediatrics.

Eligible participants were frontline physicians with independent authority to make patient admission decisions who voluntarily agreed to participate. Residents undergoing standardized residency training who had not yet obtained independent clinical decision-making authority and personnel in nonclinical positions were excluded.

The minimum sample size for the DCE was estimated using the commonly applied Johnson–Orme rule of thumb, $N>\frac{500c}{t\times a}$ (21), where c is the largest number of levels for any attribute, t is the number of choice tasks completed by each respondent, and a is the number of alternatives in each choice task. In this study, c =3, t =9, and a=3 , yielding a minimum required sample size of approximately 56 respondents. Considering the precision of parameter estimates from the mixed logit model and the need for subsequent analyses of preference heterogeneity and latent classes, we planned to recruit at least 300 eligible physicians. A total of 377 questionnaires were distributed. After quality control based on the prespecified consistency-check criterion, 339 valid questionnaires were included in the final analysis.

### 2.3 DCE design

The policy document review identified five core policy dimensions relevant to physicians’ admission decisions under the DIP payment environment: resource efficiency, technical value, service value, financial incentives, and compliance risk. These policy dimensions were then further refined through the semi-structured interviews described above into six DCE attributes, and recognizable choice scenarios were developed to reflect combinations of characteristics encountered in routine DIP-related decision-making (Table 1).

**Table 1:** DCE attributes and levels.

Attribute	Level	Definition
Clinical complexity	Low; High	Low: single diagnosis, favorable prognosis. High: two or more comorbidities, unfavorable prognosis.
Length of stay vs. institutional benchmark	At or below benchmark; Above benchmark	At or below benchmark: ≦ mean institutional length of stay. Above benchmark: > mean institutional length of stay.
Treatment technology	Conventional; Innovative	Conventional: established clinical pathway, associated with lower CMI. Innovative: novel clinical pathway, associated with higher CMI.
Share of service revenue (excluding drugs, materials, diagnostic tests)	Low; High	Low:≦ 15% of total case revenue from professional fees. High: ≧35% of total case revenue from professional fees, such as consultation or surgery.
Expected DIP margin per case	Loss; Break-even; Profit	Loss: payment < cost by ¥2,000. Break-even: payment approximately equals cost. Profit: payment > cost by ¥2,000.
Compliance risk (related to coding and treatment)	None; Moderate; High	None: fully compliant with DIP standards. Moderate: requires modification of clinical documentation to avoid financial loss, without explicit alteration of the principal diagnosis. High: requires alteration of principal diagnosis to avoid financial loss.

All DCE attribute levels were further refined during questionnaire development using an iterative qualitative approach, following an attribute-development process in which concepts are first developed and then wording is refined to accurately convey the intended meaning (22). Drawing on the practical experience of the study hospital following DIP implementation, together with input from clinicians and hospital administrators, the attribute levels and their descriptions were further refined to ensure that they could be accurately understood and recognized in routine practice. For levels involving specific numerical values, values were selected at practically recognizable and reasonable magnitudes to operationalize the corresponding categories. The finalized attribute levels were subsequently evaluated in cognitive pretesting for realism, comprehensibility, and discriminability.

A D-efficient experimental design was generated using Ngene 1.3, producing 36 choice sets that were allocated across four questionnaire versions, with nine choice tasks in each version. Each task included two unlabeled hypothetical patient alternatives and one opt-out alternative. The final experimental design achieved a D-efficiency of 100% according to the Ngene output.

Before the formal survey, cognitive pretesting was conducted among 15 physicians from the target population. The pretest primarily assessed the clarity of the attribute and level descriptions, the realism and discriminability of the scenarios, the comprehensibility of the choice tasks, and respondent burden. The final questionnaire was determined after incorporating feedback from the pretest.

The final questionnaire consisted of five sections: (1) study information and informed consent; (2) respondent characteristics; (3) definitions of the attributes and levels and an example choice task; (4) nine DCE choice tasks; and (5) one consistency-check task, which was uniformly presented as the 10th task in each questionnaire. The consistency-check task included one clearly dominant alternative and was used solely to identify questionnaires indicating insufficient attention or inconsistent responses; it was not included in the preference-model analyses. The full questionnaire is provided in Supplementary material.

### 2.4 Statistical analysis

All attribute levels were entered into the models as categorical variables, with corresponding reference levels specified. Mixed logit models were used to analyze physicians’ choice preferences and opt-out choices while accounting for the correlation arising from repeated choices within respondents. The final model specification was determined by comparing alternative random-parameter structures and jointly considering the preference heterogeneity reflected by the estimated random-parameter standard deviations and the Akaike information criterion (AIC). Models were estimated using 1,000 simulation draws. Post-estimation Wald tests were used for formal comparisons of relevant coefficients, and the relative importance of each attribute was calculated based on its utility range. Exploratory subgroup analyses were conducted to examine preference differences across physician characteristics, with joint Wald tests used to assess overall differences in preference patterns between subgroups.

The number of latent classes in the latent class logit model was determined based on the Bayesian information criterion (BIC) and consistent Akaike information criterion (CAIC). All statistical tests were two-sided, with *p* < 0.05 considered statistically significant. Statistical analyses were performed using Stata 19.0.

## 3 Results

### 3.1 Sample characteristics

The sample consisted predominantly of mid-career physicians aged 36–55 years (56.05%). Most respondents held postgraduate degrees (90.56%), reflecting the qualification structure of the study hospital. Attending physicians formed the largest professional-title group (46.61%). Internal medicine (41.89%) and surgery (30.09%) accounted for the largest departmental shares (Supplementary Table S1).

### 3.2 Aggregate preferences for patient admission

The final mixed logit model showed that all six DCE attributes were significantly associated with physicians’ admission preferences (LR chi-square = 201.47, *p* < 0.001) (Table 2). Choice sets in which the opt-out option was selected were excluded from this estimation. Expected DIP margin was the most influential attribute in admission preferences. Expected DIP loss was associated with substantially lower admission preference (*β* = −1.81, *p* < 0.001), whereas expected profit was associated with higher admission preference, but with a smaller effect size (*β* = 0.45, *p* < 0.001). A formal Wald test rejected the null hypothesis of symmetric effects ( $\beta_{\text{loss}}+\beta_{\text{profit}}=-1.36$ , SE = 0.196 *z* = −6.96, *p* < 0.001; 95% CI, −1.75 to −0.98), indicating that the negative response to expected loss was significantly greater in magnitude than the positive response to expected profit.

**Table 2:** Discrete choice model parameter estimates.

Attribute	Level	Mean (*β*)	Standard error	Random parameter SD	95% CI of *β*
Clinical complexity	High (ref: low)	−1.08***	0.11	1.21***	(−1.29, −0.87)
Length of stay vs. institutional benchmark	Above benchmark (ref: at or below benchmark)	−0.23**	0.07	0.50***	(−0.38, −0.09)
Treatment technology	Innovative (ref: conventional)	0.30***	0.07	–	(0.16, 0.43)
Share of service revenue	High (ref: low)	0.65***	0.09	0.89***	(0.47, 0.82)
Expected DIP margin per case	Loss (ref: break-even)	−1.81***	0.16	1.57***	(−2.13, −1.49)
Expected DIP margin per case	Profit (ref: break-even)	0.45***	0.10	0.74***	(0.26, 0.64)
Compliance risk	Moderate (ref: none)	−0.66***	0.11	–	(−0.88, −0.45)
Compliance risk	High (ref: none)	−0.86***	0.12	0.83***	(−1.10, −0.63)
Number of observations	5,596				
Number of respondents	339				
Log likelihood	−1426.52				
LR chi-square	201.47***				
Akaike information criterion	2881.03				
Bayesian information criterion	2973.85				

Observations refer to alternative-level observations included in the main mixed logit model. Random-parameter standard deviations (SDs) are reported only for coefficients specified as random; “–” indicates coefficients retained as fixed in the final model. The 95% CI refer to the estimated mean coefficients. Statistical significance is indicated as *p < 0.05, **p < 0.01, ***p < 0.001.

High clinical complexity was associated with lower admission preference (*β* = −1.08, *p* < 0.001). Both the moderate-and high-compliance-risk scenarios were also negatively associated with admission preference (*β* = −0.66 and −0.86, respectively; both *p* < 0.001). A higher share of service revenue was positively associated with admission preference (*β* = 0.65, *p* < 0.001). The innovative-technology scenario was positively associated with admission preference (*β* = 0.30, *p* < 0.001), whereas length of stay above the institutional benchmark was negatively associated with preference (*β* = −0.23, *p* = 0.002).

Significant random-parameter standard deviations were observed for expected loss, expected profit, clinical complexity, share of service revenue, length of stay, and high compliance risk (all *p* < 0.001), indicating substantial heterogeneity in physicians’ preferences for these attribute levels. Treatment technology and moderate compliance risk were retained as fixed coefficients in the final specification.

Relative importance scores calculated from the utility range of each attribute showed that expected DIP margin remained the most influential attribute (41.94%), followed by clinical complexity (20.11%) and compliance risk (16.01%) (Figure 1). Share of service revenue accounted for 12.03% of relative importance, whereas treatment technology (5.56%) and length-of-stay management (4.35%) contributed comparatively less.

**Figure 1:**
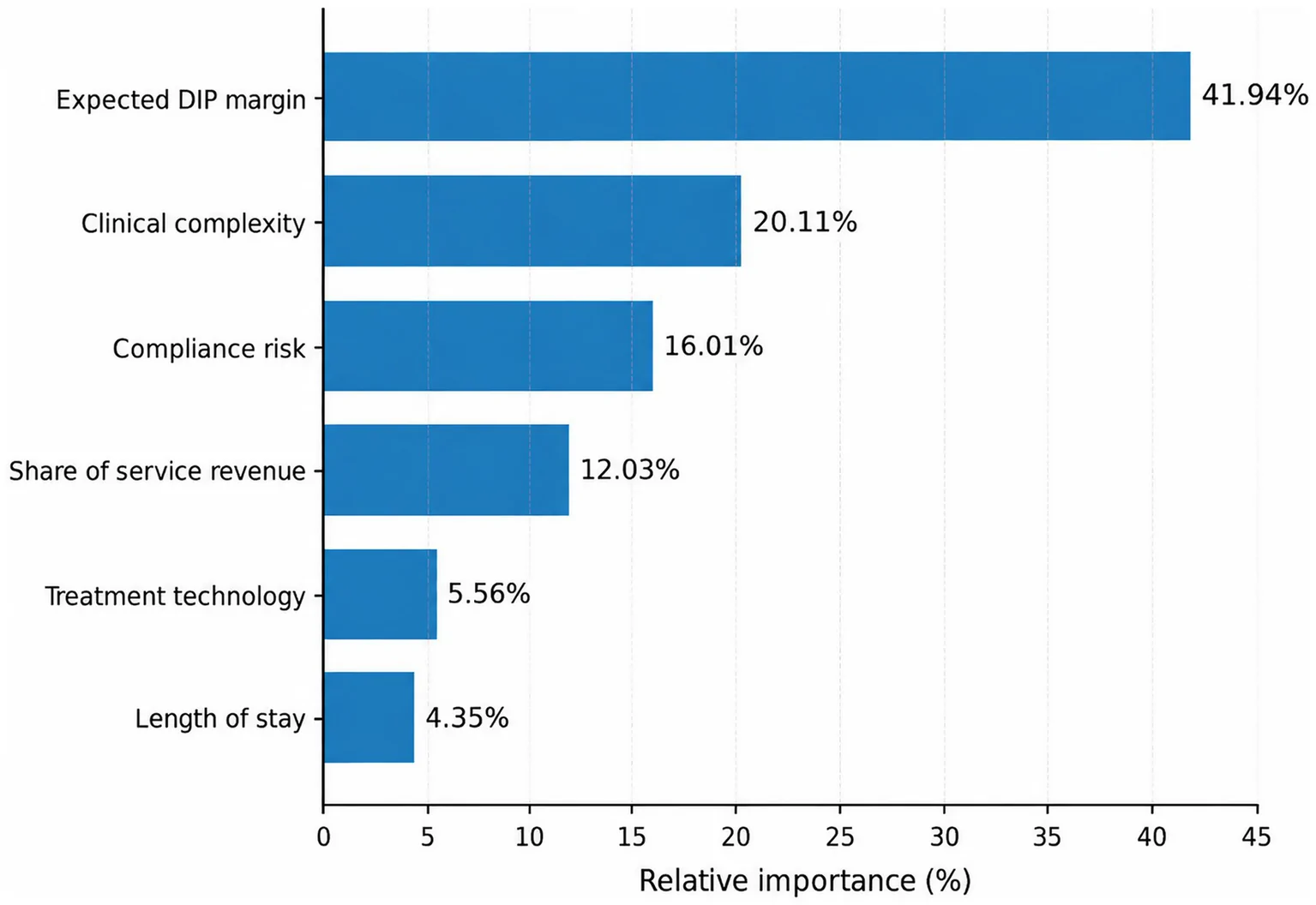
Relative importance of attributes in admission decisions.

Exploratory subgroup analyses examined three key physician characteristics: professional title, clinical role, and department. No statistically significant overall differences in preference patterns were observed across these subgroups (Supplementary Table S2).

### 3.3 Factors associated with selecting the opt-out alternative

Among the 3,051 valid choice sets completed by 339 physicians, the opt-out alternative was selected in 8.29% of choice sets. In the multivariable mixed logit model including the opt-out alternative, expected DIP loss was associated with a greater tendency to choose the opt-out option after accounting for the other DCE attributes (*β* = −2.24, *p* < 0.001). High clinical complexity (*β* = −1.31, *p* < 0.001), high compliance risk (*β* = −1.15, *p* < 0.001), moderate compliance risk (*β* = −0.78, *p* < 0.001), and above-benchmark length of stay (*β* = −0.27, *p* = 0.003) showed associations in the same direction. In contrast, a higher share of service revenue (*β* = 0.77, *p* < 0.001), expected DIP profit (*β* = 0.57, *p* < 0.001), and innovative technology (*β* = 0.33, *p* < 0.001) were associated with a lower tendency to choose the opt-out option (Supplementary Table S3).

### 3.4 Latent classes of physicians’ preference structures

To examine unobserved preference heterogeneity, latent class logit models were estimated. Models with two to seven latent classes were compared, and the five-class solution was selected based on the lowest CAIC (4746.60) and BIC (4698.60) (Supplementary Table S4). The estimated class proportions were 21.7%, 16.4%, 20.6%, 28.5%, and 12.8%, respectively. We assigned descriptive labels to summarize the preference patterns within each class. These labels refer to choices in the questionnaire and do not imply stable physician characteristics or observed clinical behavior. Class-specific estimates are presented in Figure 2, with complete results in Supplementary Table S5.

**Figure 2:**
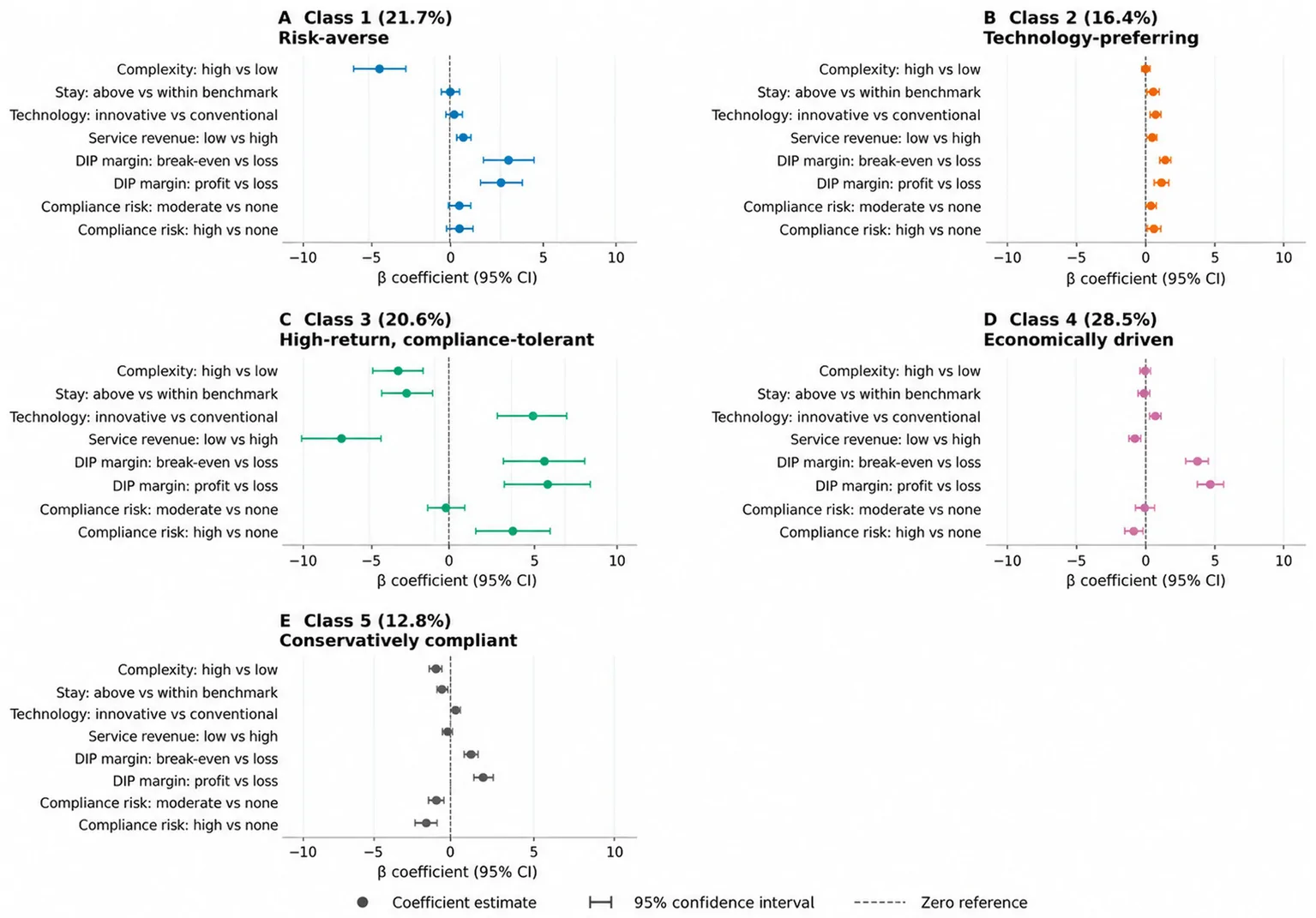
Panels **(A)–(E)** represent the five latent classes: **(A)** Class 1 (Risk-averse), **(B)** Class 2 (Technology-preferring), **(C)** Class 3 (High-return, compliance-tolerant), **(D)** Class 4 (Economically driven), and **(E)** Class 5 (Conservatively compliant). Points show coefficients and horizontal bars show 95% confidence intervals. Class proportions appear in the panel titles. Positive coefficients indicate preference for the first level compared with the reference level; negative coefficients indicate the opposite direction.

#### 3.4.1 Class 1: risk-averse (21.7%)

This class showed a negative preference for high clinical complexity. Both break-even and profitable cases were preferred to loss-making cases, and a lower service revenue share was also preferred. No statistically significant differences were observed for the length-of-stay, treatment-technology, or compliance-risk comparisons.

#### 3.4.2 Class 2: technology-preferring (16.4%)

This class preferred the innovative-technology scenario and a lower service revenue share. Both break-even and profitable cases were preferred to loss-making cases. The clinical-complexity, length-of-stay, and compliance-risk comparisons did not show statistically significant differences.

#### 3.4.3 Class 3: high-return, compliance-tolerant (20.6%)

This class showed strong preferences for financially advantageous cases and, unlike most other classes, positive utility for the high-compliance-risk scenario, while showing a strong negative preference for longer hospital stays. This class preferred a higher service revenue share and both break-even and profitable cases over loss-making cases. It also preferred the innovative-technology scenario, lower clinical complexity, and stays within the institutional benchmark. The high-compliance-risk scenario had a positive coefficient. This finding concerns the complete scenario presented in the questionnaire and should not be interpreted as willingness to engage in noncompliant practice.

#### 3.4.4 Class 4: economically driven (28.5%)

This class preferred both break-even and profitable cases over loss-making cases, alongside the innovative-technology scenario and a higher service revenue share. The high-compliance-risk scenario had a negative coefficient. The clinical-complexity, length-of-stay, and moderate-compliance-risk comparisons were not statistically significant.

#### 3.4.5 Class 5: conservatively compliant (12.8%)

This class showed negative preferences for high clinical complexity, stays above the institutional benchmark, and moderate- and high-compliance-risk scenarios. It also preferred the innovative-technology scenario and both break-even and profitable cases over loss-making cases. The service-revenue-share comparison was not statistically significant.

## 4 Discussion

### 4.1 Financial incentives, loss aversion, and preference heterogeneity

Financial incentives were strongly associated with admission preferences, with a clear asymmetry between expected losses and gains. In the mixed logit model, the formal coefficient comparison showed that the negative utility associated with expected loss was significantly greater in magnitude than the positive utility associated with expected profit. This asymmetric response is consistent with the behavioral economics concept of loss aversion (23).

Compared with fee-for-service payment, DIP-style point-based payment under a global budget frames each case as a financial unit. In such a setting, physicians and hospitals may become especially sensitive to cases expected to generate losses. This mechanism is consistent with the logic of risk selection in payment systems that transfer financial risk to providers, in which providers may avoid high-cost or complex cases when financial risk is not adequately adjusted (24, 25).

Preferences for financial attributes varied across physicians. Latent class analysis revealed five distinct preference profiles. The economically driven and high-return, compliance-tolerant groups both showed strong preferences for financially favorable cases; however, the former showed negative utility for the high-compliance-risk scenario, whereas the latter showed positive utility for this scenario. In contrast, the risk-averse and conservatively compliant groups prioritized avoiding financial or regulatory risk. This heterogeneity suggests that physicians’ preference patterns may reflect not only responses to external incentives (26), but also unmeasured individual- or organizational-level factors, potentially including risk preference and professional motivation (27, 28). However, exploratory subgroup analyses did not identify statistically significant overall differences in preference patterns by professional title, clinical role, or department. These findings suggest that the observed heterogeneity is not readily explained by these broad observable professional characteristics.

### 4.2 Regulatory risk and admission preferences

Compliance risk was the third most influential attribute after expected DIP margin and clinical complexity, suggesting that regulatory considerations are salient in physicians’ admission preferences under DIP. Similar to audit mechanisms in Germany (29) and DRG legislation in the United States (30), DIP involves data-driven audits, inspections, and penalties (31, 32). These mechanisms may make coding and treatment compliance salient professional and financial concerns and may also intensify the dual-agency dilemma in which physicians and managers must balance patient welfare with organizational financial performance (33).

The multivariable opt-out analysis showed that both moderate- and high-compliance-risk scenarios were associated with a greater tendency to select the opt-out option after accounting for the other DCE attributes. This pattern may reflect physicians’ responses to the bundled clinical, financial, and regulatory information presented across choice tasks. Such responses are consistent with multi-task incentive distortion, where attention shifts toward the dimensions most strongly rewarded or penalized (34–36). However, because compliance risk was operationalized as a composite scenario, the present design does not allow the contribution of individual components to be isolated. These findings nevertheless suggest that payment and regulatory design should be considered jointly rather than as separate policy instruments.

### 4.3 Balancing operational efficiency and technical value

The study also found that physicians preferred cases with within-benchmark length of stay, the innovative-technology scenario, and a higher share of service revenue. These preferences broadly align with DIP’s stated goals of improving operational efficiency and recognizing technical labor (37, 38).

At the same time, incentive alignment can create new distortions. Preference for the innovative-technology scenario or high-service-revenue cases may be consistent with clinical value when clinically appropriate, but such incentives may also create risks of technology overuse or upcoding if insufficiently governed (14, 39). Therefore, the technology-preferring and high-return, compliance-tolerant classes identified in this study warrant managerial attention. These findings support the need for payment designs that combine cost control with clinical-pathway standards, quality monitoring, and safeguards against low-value care.

### 4.4 Policy and management implications

First, at the payment-design level, value-oriented incentives could be further strengthened. International experience suggests that no single payment method can fully balance cost containment, quality, and equity (40), while value-based payment, pay-for-performance, and bundled payment models may help better align reimbursement with quality, efficiency, and outcomes (41). Based on the preference patterns observed in this study, risk adjustment for patients with high clinical complexity or multiple comorbidities could be further refined, supplementary compensation could be considered for unusually high-cost or severely ill patients, and recurrent loss-making case groups could be identified to assess whether current point values adequately reflect their actual resource use. These measures may help reduce excessive sensitivity to case-level financial losses.

Second, at the regulatory level, a balance should be sought between risk control and upstream guidance. Excessive reliance on retrospective, penalty-driven audits may increase risk avoidance without necessarily improving clinical value (42). Regulatory strategies could combine risk identification, real-time or pre-discharge alerts, standardized coding guidance, department-specific training, and consultative feedback. For first-time, non-malicious documentation problems, corrective feedback could be prioritized, with regulatory responses escalated according to the level of risk. Such an approach may improve the timeliness and targeting of oversight while maintaining regulatory accountability.

Third, at the hospital-management level, cost-control objectives should be better aligned with quality-of-care goals. Practices such as disease-specific cost accounting, clinical pathway optimization, and performance evaluation reform may help translate payment-policy objectives into clinical management (43). Hospitals could establish department-level DIP cost-accounting and visual monitoring tools, regularly review high-cost and recurrent loss-making cases, and continuously optimize clinical pathways for complex patients. Internal performance evaluation should also avoid excessive reliance on departmental financial surplus and incorporate indicators of clinical appropriateness, patient safety, and quality of care.

### 4.5 Limitations

This study has several limitations. First, it was conducted in a single tertiary hospital using a cross-sectional design. Physicians’ preferences may reflect both the local DIP implementation environment and hospital-level governance, and the absence of a pre-policy baseline or comparison group precludes causal inference. The findings should therefore be interpreted as context-specific rather than as evidence of the causal effect of DIP reform.

Second, the DCE elicited stated preferences in hypothetical admission scenarios rather than observed clinical behavior. Responses were not linked to actual admission records, and social desirability bias may have influenced physicians’ choices, particularly for financially or ethically sensitive attributes. The extent to which these preferences translate into real-world behavior therefore remains uncertain.

Third, some DCE attributes were operationalized as composite scenarios. In particular, the compliance-risk levels may contain conceptually overlapping elements; therefore, the estimated coefficients should be interpreted as preferences for the scenarios as presented rather than as independent effects of individual components.

## 5 Conclusion

In this discrete choice experiment conducted in a Chinese tertiary hospital operating under the Diagnosis-Intervention Packet payment system, physicians’ admission preferences reflected trade-offs across financial, clinical, operational, and regulatory attributes. Expected case margin was the most influential attribute, with a stronger negative response to expected loss than the positive response to expected profit, consistent with loss aversion. Substantial preference heterogeneity was also observed. These findings provide context-specific evidence on physician decision trade-offs within a DIP implementation setting and may inform local payment and hospital-management strategies. Multicenter and longitudinal studies linking stated preferences with actual admission behavior are needed to evaluate their generalizability and real-world relevance.

## Data Availability

The raw data supporting the conclusions of this article will be made available by the authors, without undue reservation.
