# Supplementary material for: Physician admission preferences under the Diagnosis-Intervention Packet payment system: a discrete choice experiment in a Chinese tertiary hospital
Source: Front Public Health. 2026 Oct 22;14:1946819. doi: 10.3389/fpubh.2026.1946819 (PMC13735536; doi:10.3389/fpubh.2026.1946819)
Supplement: Supplementary file 1 [file Data_Sheet_1.docx]

Supplementary Material

# 1.Policy document review

A total of 16 policy documents related to Diagnosis-Intervention Packet (DIP) payment reform were identified through a review of the official websites of the National Health Commission, the National Healthcare Security Administration, provincial and municipal health administrative departments, and governments at various levels. The documents spanned January 2016 to December 2025 . Using NVivo 11 software, the texts of these policy documents were coded word-by-word and sentence-by-sentence. Codes were compared, refined, and categorized, ultimately forming five policy dimensions .

**Policy documents reviewed for DIP payment reform**

| No. | Region | Document Title | Issuance Date | Issuing Authority |
| --- | --- | --- | --- | --- |
| 1 | National | Opinions of the State Council on Integrating the Basic Medical Insurance Systems for Urban and Rural Residents | 3-Jan-16 | State Council |
| 2 | National | Regulations on the National Social Security Fund | 10-Mar-16 | National Healthcare Security Administration |
| 3 | National | Outline of the "Healthy China 2030" Plan Issued by the CPC Central Committee and the State Council (2016) | 25-Oct-16 | State Council |
| 4 | National | Notice of the State Council on Issuing the Plan for Deepening the Reform of the Medical and Health System during the 13th Five-Year Plan Period | 27-Dec-16 | State Council |
| 5 | National | Notice of the General Office of the State Council on Issuing the Pilot Scheme for Merging the Implementation of Maternity Insurance and the Basic Medical Insurance for Employees | 19-Jan-17 | State Council |
| 6 | National | Guiding Opinions of the General Office of the State Council on Further Deepening the Reform of Payment Methods for Basic Medical Insurance | 20-Jun-17 | State Council |
| 7 | National | Notice on Issuing the National Pilot Technical Specifications and Grouping Scheme for Diagnosis Related Groups (DRG) Payment | 24-Oct-19 | National Healthcare Security Administration |
| 8 | National | Notice of the General Office of the National Healthcare Security Administration on Issuing the Detailed Grouping Scheme for Healthcare Security Diagnosis Related Groups (CHS-DRG) (Version 1.0) | 18-Jun-20 | National Healthcare Security Administration |
| 9 | National | Notice of the General Office of the National Healthcare Security Administration on Issuing the Pilot Work Plan for Regional Global Budget Based on Points and Payment Based on Disease Score | 14-Oct-20 | National Healthcare Security Administration |
| 10 | National | Notice of the General Office of the National Healthcare Security Administration on Issuing the Technical Specifications for National Healthcare Security Payment Based on Disease Score (DIP) and the DIP Disease Catalog Library (Version 1.0) | 20-Nov-20 | National Healthcare Security Administration |
| 11 | National | Notice of the General Office of the National Healthcare Security Administration on Issuing the Operational Management Regulations (Trial) for Healthcare Security Payment Based on Disease Score (DIP) | 15-Jul-21 | National Healthcare Security Administration |
| 12 | National | Notice of the General Office of the State Council on Issuing the "14th Five-Year" Plan for National Healthcare Security | 29-Sep-21 | State Council |
| 13 | National | Notice of the National Healthcare Security Administration on Issuing the Three-Year Action Plan for DRG/DIP Payment Method Reform | 26-Nov-21 | National Healthcare Security Administration |
| 14 | National | Notice of the General Office of the National Healthcare Security Administration on Issuing the 2.0 Version Grouping Scheme for Payment by DRG and Disease Score and on Further Promoting Related Work | 17-Jul-24 | National Healthcare Security Administration |
| 15 | National | Notice of the General Office of the National Healthcare Security Administration on Issuing the "Healthcare Security Operational Management Regulations for Payment by Disease (2025 Edition)" | 27-Jan-25 | National Healthcare Security Administration |
| 16 | National | Notice of the National Healthcare Security Administration on Issuing the "Interim Measures for the Management of Healthcare Security Payment by Disease" | 11-Aug-25 | National Healthcare Security Administration |

**Coding framework derived from policy document review**

| Selective Coding  (Core Dimensions) | Axial Coding  (Categories) | Open Coding  (Initial Concepts) | Original Policy Entries  (Direct Quotations) |
| --- | --- | --- | --- |
| Resource Efficiency | C1. Fund Budgeting and Balancing Principles | A1. System design follows the principles of moderate coverage and revenue-expenditure balance | "Follow the principles of moderate coverage and revenue-expenditure balance, and equalize urban-rural benefit levels." |
|  |  | A4. Determining global budget based on the principle of 'expenditure based on revenue' | "Pooling regions should reasonably determine medical insurance global budget targets according to the principles of expenditure based on revenue, revenue-expenditure balance, and slight surplus." |
|  |  | A6. Improving global budget management methods to enhance scientificity of targets | "Improve global budget management methods to enhance the scientificity and rationality of budget control targets." |
|  |  | A36. Improving the multi-level healthcare security system | "Improve the multi-level healthcare security system with basic medical insurance as the main body, supplemented by other forms of supplementary insurance and commercial health insurance." |
|  |  | A37. Maternity insurance fund merged with employee basic medical insurance fund for unified collection | "The maternity insurance fund is merged into the employee basic medical insurance fund for unified collection." |
|  |  | A38. Social security fund operation adheres to principles of safety, profitability, and long-term orientation | "The National Council for Social Security Fund, when investing and operating the national social security fund, shall adhere to the principles of safety, profitability, and long-term orientation." |
|  | C2. Fund and Resource Utilization Efficiency | A2. Improving medical insurance fund use efficiency and promoting rational resource utilization | "Continuously improve the efficiency of medical insurance fund use, focus on safeguarding the basic medical needs of insured persons, and promote the rational use of healthcare resources." |
|  | C3. Payment Method Design and Structure | A3. Comprehensively implementing a diversified composite medical insurance payment method primarily based on payment by disease | "Comprehensively implement a diversified composite medical insurance payment method primarily based on payment by disease." |
|  |  | A5. Replacing individual institution global budget control with regional point-based payment | "Instead of specifying detailed global budget control targets for individual medical institutions, convert payment units such as services, diseases, and bed-days into certain points... At year-end, payment is made to each medical institution based on its actual total points according to the services provided." |
|  |  | A39. Applying a unified CHS-DRG grouping system | "Apply a unified CHS-DRG grouping system." |
|  |  | A40. Widespread implementation of diversified payment methods primarily based on payment by disease | "Widely implement diversified composite medical insurance payment methods primarily based on payment by disease across the country." |
|  | C4. Cost Control and Behavioral Guidance | A7. Stimulating the endogenous motivation of medical institutions to standardize behavior, control costs, and reasonably admit and refer patients | "Stimulate the endogenous motivation of medical institutions to standardize behavior, control costs, and reasonably admit and refer patients." |
|  |  | A8. Controlling unreasonable growth of medical expenses in public hospitals | "Control unreasonable growth of medical expenses in public hospitals." |
| Technical Value | C5. Recognition of Technical Complexity Value | A9. Allowing special case review for complex/special cases | "For cases unsuitable for standard DRG/DIP payment due to long hospital stays, high medical costs, use of new drugs/technologies, complex/critical conditions, or multi-disciplinary treatment, medical institutions may independently apply for special case review." |
|  |  | A10. Providing special payment support for new drugs/technologies in the initial application phase | "Explore exclusion mechanisms, providing support for eligible new drugs and technologies in the initial application phase through fee-for-service payment or weight (point) bonuses." |
|  | C6. Reflection of Specific Service Value | A11. Exploring characteristic payment methods for Traditional Chinese Medicine and encouraging its use | "Explore payment methods suitable for the characteristics of TCM services, and encourage the provision and use of appropriate TCM services." |
|  |  | A12. Exploring equal payment for equal disease and efficacy between TCM and Western medicine | "Explore medical insurance payment methods reflecting TCM characteristics... Encourage implementing equal payment for equal disease and efficacy between TCM and Western medicine." |
|  |  | A13. DIP design reflects the labor value of medical personnel | "DIP, supported by big data... reflects the labor value of medical personnel." |
|  | C7. Standardization of Diagnosis and Treatment Process | A14. Increasing transparency of diagnosis and treatment behavior | "Increase the transparency of diagnosis and treatment behavior." |
|  | C9. Economic Incentives and Risk Sharing | A15. Implementing clinical pathway management | "Implement clinical pathway management." |
|  |  | A16. Promoting mutual recognition of examination and test results | "Promote mutual recognition of medical examination and test results among peer medical institutions to reduce duplicate tests." |
| Economic Incentives |  | A20. Establishing incentive and risk-sharing mechanisms of 'surplus retention and reasonable over-expenditure sharing' | "Establish and improve negotiation mechanisms, and incentive and risk-sharing mechanisms of 'surplus retention and reasonable over-expenditure sharing'." |
|  |  | A21. Requiring implementation of surplus retention and over-expenditure sharing mechanisms | "Implement the mechanisms of 'surplus retention and reasonable over-expenditure sharing' as per agreements, ensuring all agreed-upon budgets are used for settlement." |
|  | C10. Fund Prepayment and Settlement Arrangements | A22. Encouraging fund prepayment to alleviate institutional financial pressure | "Encourage alleviating medical institutions' financial pressure through fund prepayment... prepay approximately one month's advance payment to designated medical institutions." |
|  |  | A23. Stipulating prepayment of medical insurance funds to alleviate pressure | "Fund prepayment. Pooling regions may prepay a portion of medical insurance funds to designated medical institutions as per national regulations to alleviate their financial operational pressure." |
|  | C11. Application of Performance Assessment Results | A24. Linking assessment results with medical insurance fund payment | "Link assessment results with medical insurance fund payment." |
| Compliance Risk | C12. Monitoring and Handling of Violations | A25. Focusing on and addressing violations like upcoding in DIP payment | "Focus on and propose specific measures to address violations such as upcoding and mismatched diagnoses and procedures occurring in DIP payment by designated medical institutions." |
|  |  | A26. Preventing behaviors like splitting hospitalizations and refusing patients | "Prevent potential behaviors such as service reduction, upcoding, splitting hospitalizations, and refusing patients." |
|  | C13.Intelligent Monitoring and Data Supervision | A27. Establishing comprehensive, whole-process big data intelligent monitoring | "Comprehensively establish an intelligent monitoring system... achieving a shift in fund supervision from manual sampling review to comprehensive, whole-process, all-link intelligent monitoring using big data." |
|  |  | A28. Promoting full coverage of intelligent review and regular data screening | "Promote full coverage of intelligent review and regularly conduct screening and analysis of medical insurance data." |
|  | C14. Data Quality and Standardization Requirements | A29. Requiring strict adherence to norms for filling out settlement list information | "Clearly stipulate that designated medical institutions shall strictly fill out diagnosis, treatment, and cost information for inpatient services according to the healthcare security fund settlement list and its filling specifications." |
|  |  | A30. Requiring unified use of medical insurance information business coding standards | "All pilot cities shall uniformly use medical insurance information business coding standards for disease diagnosis, surgical operations, medical service items, drugs, medical consumables, etc." |
|  | C15. Credit Evaluation and Comprehensive Disciplinary Measures | A31. Establishing a closed-loop, full-chain credit supervision system | "Improve the medical security credit management system, forming a closed-loop, full-chain credit supervision encompassing credit commitment, credit evaluation, information sharing, result disclosure, result application, and credit repair." |
|  |  | A32. Exploring extending supervision to the service behavior of medical personnel | "Actively explore effective ways to extend medical insurance supervision to the medical service behavior of healthcare personnel." |
|  | C16. Negotiation and Dispute Resolution Mechanisms | A33. Establishing a DIP dispute resolution mechanism | "Establish a DIP dispute resolution mechanism, resolving disputes raised by designated medical institutions according to the principles of 'fairness and justice, objectivity and reasonableness, multi-party participation, and timely handling'." |
|  |  | A34. Establishing a negotiation mechanism for core payment elements | "Each pooling region shall establish and improve negotiation mechanisms concerning core payment elements such as global budget, weights, scores, and adjustment coefficients." |
| Service Value | C8. Quality, Safety, and Performance Managemen t | A35. Unifying and strengthening agreement management for designated institutions | "Unify the management measures for designated institutions under urban and rural residents' medical insurance, and strengthen designated service agreement management." |
|  |  | A17. Shifting supervision focus to dual control of cost and quality | "Strengthen medical insurance supervision over medical behavior... shift the supervision focus from medical cost control to dual control of medical costs and medical quality." |
|  |  | A18. Linking quality assessment results with fund payment | "Strengthen medical service quality control... link assessment results with medical insurance fund payment." |
|  |  | A19. Emphasizing medical record management as the core and requiring quality improvement | "Strengthening medical record management is the core of DIP grouping. Guide medical institutions to effectively strengthen in-house medical record management and improve its quality." |

# 2 Survey questionnaire

**Clinician preferences in patient admission decisions under the Diagnosis-Intervention Packet payment reform: survey questionnaire**

*Version A - one of four parallel versions*

**Informed Consent Form**

Dear Clinician,

Greetings. We are conducting a study to understand the preferences and decision-making trade-offs clinicians consider when admitting patients under the Diagnosis-Intervention Packet (DIP) payment reform. The following information is provided to help you decide whether to participate.

This study collects your views through an anonymous questionnaire. There are no right or wrong answers. If you agree to participate, you will complete one questionnaire, estimated to take 3-5 minutes.

Your responses will be used solely for aggregate analysis and will not be used for any form of personal evaluation. Your participation is voluntary, and you may decline participation or withdraw at any time without any adverse consequences.

If you understand the above information and agree to participate, please proceed with the questionnaire.

**A. Basic information**

A1. Gender: □ Male □ Female

A2. Age: □ Under 25 □ 26-35 □ 36-55 □ 56+

A3. Educational background: □ Below Bachelor's degree □ Bachelor's degree □ Master's degree □ Doctorate

A4. Professional title: □ Resident physician □ Attending physician □ Associate chief physician □ Chief physician

A5. Role: □ Regular physician □ Head of diagnostic and treatment team □ Department director

A6. Department: □ Internal medicine □ Surgery □ Obstetrics and gynecology □ Pediatrics □ Integrative medicine/Other ____

A7. Years of experience: □ <5 years □ 5-10 years □ 11-20 years □ >20 years

A8. Compensation level (annual after-tax income): □ <150,000 □ 150,000-250,000 □ >250,000 CNY

**Scenario simulation: clinician patient admission decision**

The following section presents 10 choice tasks. Tasks 1-9 were used for analysis, and Task 10 was used as a consistency-check item. In each task, please select the patient you would be more likely to admit based on your clinical experience. You may also choose the opt-out option.

**Attribute definitions and explanations**

| **Attribute** | **Level** | **Definition** |
| --- | --- | --- |
| Clinical complexity | Low; High | Low: single diagnosis, favorable prognosis. High: two or more comorbidities, unfavorable prognosis. |
| Length of stay vs. institutional benchmark | At or below benchmark; Above benchmark | At or below benchmark: <= mean institutional length of stay. Above benchmark: > mean institutional length of stay. |
| Treatment technology | Conventional; Innovative | Conventional: established clinical pathway, associated with lower CMI. Innovative: novel clinical pathway, associated with higher CMI. |
| Share of service revenue (excluding drugs, materials, diagnostic tests) | Low; High | Low: <=15% of total case revenue from professional fees. High: >=35% of total case revenue from professional fees, such as consultation or surgery. |
| Expected DIP margin per case | Loss; Break-even; Profit | Loss: payment < cost by ¥2,000. Break-even: payment approximately equals cost. Profit: payment > cost by ¥2,000. |
| Compliance risk (related to coding and treatment) | None; Moderate; High | None: fully compliant with DIP standards. Moderate: requires modification of clinical documentation to avoid financial loss. High: requires alteration of principal diagnosis to avoid financial loss. |

**Plan One**

| **Attribute** | **Patient A** | **Patient B** |
| --- | --- | --- |
| Clinical complexity | Low (single diagnosis, favorable prognosis) | High (>=2 comorbidities, unfavorable prognosis) |
| Length of stay vs. institutional benchmark | At or below benchmark (<= benchmark) | Above benchmark (> benchmark) |
| Treatment technology | Conventional (mature protocol, lower CMI) | Innovative (novel protocol, higher CMI) |
| Share of service revenue | High (>=35%) | Low (<=15%) |
| Expected DIP margin per case | Loss (payment < cost by ¥2,000) | Profit (payment > cost by ¥2,000) |
| Compliance risk | None (fully compliant to meet cost target) | Moderate (requires documentation adjustment to avoid loss) |
| Your choice? | □ Patient A □ Patient B □ Opt-out | |

**Plan Two**

| **Attribute** | **Patient A** | **Patient B** |
| --- | --- | --- |
| Clinical complexity | Low (single diagnosis, favorable prognosis) | High (>=2 comorbidities, unfavorable prognosis) |
| Length of stay vs. institutional benchmark | Above benchmark (> benchmark) | At or below benchmark (<= benchmark) |
| Treatment technology | Innovative (novel protocol, higher CMI) | Conventional (mature protocol, lower CMI) |
| Share of service revenue | High (>=35%) | Low (<=15%) |
| Expected DIP margin per case | Loss (payment < cost by ¥2,000) | Profit (payment > cost by ¥2,000) |
| Compliance risk | Moderate (requires documentation adjustment to avoid loss) | High (requires modifying principal diagnosis to avoid loss) |
| Your choice? | □ Patient A □ Patient B □ Opt-out | |

**Plan Three**

| **Attribute** | **Patient A** | **Patient B** |
| --- | --- | --- |
| Clinical complexity | Low (single diagnosis, favorable prognosis) | High (>=2 comorbidities, unfavorable prognosis) |
| Length of stay vs. institutional benchmark | At or below benchmark (<= benchmark) | Above benchmark (> benchmark) |
| Treatment technology | Conventional (mature protocol, lower CMI) | Innovative (novel protocol, higher CMI) |
| Share of service revenue | High (>=35%) | Low (<=15%) |
| Expected DIP margin per case | Profit (payment > cost by ¥2,000) | Break-even (payment approximately equals cost) |
| Compliance risk | Moderate (requires documentation adjustment to avoid loss) | High (requires modifying principal diagnosis to avoid loss) |
| Your choice? | □ Patient A □ Patient B □ Opt-out | |

**Plan Four**

| **Attribute** | **Patient A** | **Patient B** |
| --- | --- | --- |
| Clinical complexity | High (>=2 comorbidities, unfavorable prognosis) | Low (single diagnosis, favorable prognosis) |
| Length of stay vs. institutional benchmark | Above benchmark (> benchmark) | At or below benchmark (<= benchmark) |
| Treatment technology | Innovative (novel protocol, higher CMI) | Conventional (mature protocol, lower CMI) |
| Share of service revenue | High (>=35%) | Low (<=15%) |
| Expected DIP margin per case | Profit (payment > cost by ¥2,000) | Break-even (payment approximately equals cost) |
| Compliance risk | None (fully compliant to meet cost target) | Moderate (requires documentation adjustment to avoid loss) |
| Your choice? | □ Patient A □ Patient B □ Opt-out | |

**Plan Five**

| **Attribute** | **Patient A** | **Patient B** |
| --- | --- | --- |
| Clinical complexity | High (>=2 comorbidities, unfavorable prognosis) | Low (single diagnosis, favorable prognosis) |
| Length of stay vs. institutional benchmark | At or below benchmark (<= benchmark) | Above benchmark (> benchmark) |
| Treatment technology | Innovative (novel protocol, higher CMI) | Conventional (mature protocol, lower CMI) |
| Share of service revenue | Low (<=15%) | High (>=35%) |
| Expected DIP margin per case | Break-even (payment approximately equals cost) | Loss (payment < cost by ¥2,000) |
| Compliance risk | Moderate (requires documentation adjustment to avoid loss) | High (requires modifying principal diagnosis to avoid loss) |
| Your choice? | □ Patient A □ Patient B □ Opt-out | |

**Plan Six**

| **Attribute** | **Patient A** | **Patient B** |
| --- | --- | --- |
| Clinical complexity | High (>=2 comorbidities, unfavorable prognosis) | Low (single diagnosis, favorable prognosis) |
| Length of stay vs. institutional benchmark | At or below benchmark (<= benchmark) | Above benchmark (> benchmark) |
| Treatment technology | Conventional (mature protocol, lower CMI) | Innovative (novel protocol, higher CMI) |
| Share of service revenue | Low (<=15%) | High (>=35%) |
| Expected DIP margin per case | Loss (payment < cost by ¥2,000) | Profit (payment > cost by ¥2,000) |
| Compliance risk | High (requires modifying principal diagnosis to avoid loss) | None (fully compliant to meet cost target) |
| Your choice? | □ Patient A □ Patient B □ Opt-out | |

**Plan Seven**

| **Attribute** | **Patient A** | **Patient B** |
| --- | --- | --- |
| Clinical complexity | Low (single diagnosis, favorable prognosis) | High (>=2 comorbidities, unfavorable prognosis) |
| Length of stay vs. institutional benchmark | Above benchmark (> benchmark) | At or below benchmark (<= benchmark) |
| Treatment technology | Conventional (mature protocol, lower CMI) | Innovative (novel protocol, higher CMI) |
| Share of service revenue | Low (<=15%) | High (>=35%) |
| Expected DIP margin per case | Break-even (payment approximately equals cost) | Loss (payment < cost by ¥2,000) |
| Compliance risk | High (requires modifying principal diagnosis to avoid loss) | None (fully compliant to meet cost target) |
| Your choice? | □ Patient A □ Patient B □ Opt-out | |

**Plan Eight**

| **Attribute** | **Patient A** | **Patient B** |
| --- | --- | --- |
| Clinical complexity | Low (single diagnosis, favorable prognosis) | High (>=2 comorbidities, unfavorable prognosis) |
| Length of stay vs. institutional benchmark | At or below benchmark (<= benchmark) | Above benchmark (> benchmark) |
| Treatment technology | Innovative (novel protocol, higher CMI) | Conventional (mature protocol, lower CMI) |
| Share of service revenue | Low (<=15%) | High (>=35%) |
| Expected DIP margin per case | Profit (payment > cost by ¥2,000) | Break-even (payment approximately equals cost) |
| Compliance risk | Moderate (requires documentation adjustment to avoid loss) | High (requires modifying principal diagnosis to avoid loss) |
| Your choice? | □ Patient A □ Patient B □ Opt-out | |

**Plan Nine**

| **Attribute** | **Patient A** | **Patient B** |
| --- | --- | --- |
| Clinical complexity | Low (single diagnosis, favorable prognosis) | High (>=2 comorbidities, unfavorable prognosis) |
| Length of stay vs. institutional benchmark | At or below benchmark (<= benchmark) | Above benchmark (> benchmark) |
| Treatment technology | Conventional (mature protocol, lower CMI) | Innovative (novel protocol, higher CMI) |
| Share of service revenue | High (>=35%) | Low (<=15%) |
| Expected DIP margin per case | Break-even (payment approximately equals cost) | Loss (payment < cost by ¥2,000) |
| Compliance risk | None (fully compliant to meet cost target) | Moderate (requires documentation adjustment to avoid loss) |
| Your choice? | □ Patient A □ Patient B □ Opt-out | |

**Plan Ten***

| **Attribute** | **Patient A** | **Patient B** |
| --- | --- | --- |
| Clinical complexity | Low (single diagnosis, favorable prognosis) | High (>=2 comorbidities, unfavorable prognosis) |
| Length of stay vs. institutional benchmark | At or below benchmark (<= benchmark) | Above benchmark (> benchmark) |
| Treatment technology | Innovative (novel protocol, higher CMI) | Conventional (mature protocol, lower CMI) |
| Share of service revenue | High (>=35%) | Low (<=15%) |
| Expected DIP margin per case | Profit (payment > cost by ¥2,000) | Loss (payment < cost by ¥2,000) |
| Compliance risk | None (fully compliant to meet cost target) | High (requires modifying principal diagnosis to avoid loss) |
| Your choice? | □ Patient A □ Patient B □ Opt-out | |

*Note: * denotes the consistency-check choice set.*

# 3. Supplementary Tables

# Supplementary Table S1. Characteristics of study participants (n = 339)

| Characteristic | Category | n | % |
| --- | --- | --- | --- |
| Gender | Male | 195 | 57.52 |
|  | Female | 144 | 42.48 |
| Age (years) | 26-35 | 139 | 41.00 |
|  | 36-55 | 190 | 56.05 |
|  | >=56 | 10 | 2.95 |
| Educational attainment | Below bachelor’s degree | 1 | 0.29 |
|  | Bachelor’s degree | 31 | 9.14 |
|  | Master’s degree | 291 | 85.84 |
|  | Doctoral degree | 16 | 4.72 |
| Clinical experience (years) | <5 | 67 | 19.76 |
|  | 5-10 | 125 | 36.87 |
|  | 11-20 | 87 | 25.66 |
|  | >20 | 60 | 17.70 |
| Professional title | Resident physician | 69 | 20.35 |
|  | Attending physician | 158 | 46.61 |
|  | Associate chief physician | 78 | 23.01 |
|  | Chief physician | 34 | 10.03 |
| Clinical role | Staff physician | 256 | 75.52 |
|  | Clinical team leader | 46 | 13.57 |
|  | Department director | 37 | 10.91 |
| Annual after-tax income (CNY) | <150,000 | 229 | 67.55 |
|  | 150,000-250,000 | 94 | 27.73 |
|  | >250,000 | 16 | 4.72 |
| Department | Internal medicine | 142 | 41.89 |
|  | Surgery | 102 | 30.09 |
|  | Obstetrics and gynecology | 13 | 3.83 |
|  | Pediatrics | 44 | 12.98 |
|  | Others | 38 | 11.21 |

# Supplementary Table S2. Exploratory interaction analyses of preference heterogeneity across physician subgroups

| Characteristic | Category | Joint Wald χ² (df) | P value |
| --- | --- | --- | --- |
| Professional title | Resident physician | 23.88 (24) | 0.469 |
|  | Attending physician |  |  |
|  | Associate chief physician |  |  |
| Clinical role | Staff physician | 25.87 (16) | 0.056 |
|  | Clinical team leader |  |  |
|  | Department director |  |  |
| Department | Internal medicine | 45.47 (32) | 0.058 |
|  | Surgery |  |  |
|  | Obstetrics and gynecology |  |  |
|  | Pediatrics |  |  |
|  | Others |  |  |

*Note: Joint Wald χ², joint Wald chi-square statistic; df, degrees of freedom. P < 0.05 was considered statistically significant.*

**Supplementary Table S3. Multivariable mixed logit analysis of opt-out decisions**

| Attribute | Level | Mean (β) | Standard error | Random parameter SD | 95% CI |
| --- | --- | --- | --- | --- | --- |
| Opt-out alternative | ASC | −3.06*** | 0.16 | — | (−3.37, −2.75) |
| Clinical complexity | High (ref: low) | −1.31*** | 0.12 | 1.56*** | (−1.55, −1.07) |
| Length of stay vs. institutional benchmark | Above benchmark (ref: at or below benchmark) | −0.27** | 0.09 | 0.92*** | (−0.44, −0.09) |
| Treatment technology | Innovative (ref: conventional) | 0.33*** | 0.08 | — | (0.18, 0.49) |
| Share of service revenue | High (ref: low) | 0.77*** | 0.10 | 1.23*** | (0.57, 0.97) |
| Expected DIP margin per case | Loss (ref: break-even) | −2.24*** | 0.19 | 2.08*** | (−2.61, −1.88) |
| Expected DIP margin per case | Profit (ref: break-even) | 0.57*** | 0.10 | 0.98*** | (0.36, 0.77) |
| Compliance risk | Moderate (ref: none) | −0.78*** | 0.12 | — | (−1.00, −0.55) |
| Compliance risk | High (ref: none) | −1.15*** | 0.14 | 1.35*** | (−1.43, −0.88) |

**Model statistics:** 339 respondents; 3,051 choice tasks; 9,153 alternative-level observations; log likelihood = −2127.19; LR χ²(6) = 481.47, p < 0.001; 1,000 simulation draws.

*Note: ASC, alternative-specific constant; CI, confidence interval; DIP, Diagnosis-Intervention Packet; SD, standard deviation. The opt-out alternative was included together with the two patient alternatives, and all DCE attributes were entered simultaneously. DCE attribute variables were coded as 0 for the opt-out alternative. Negative attribute coefficients indicate that the corresponding patient characteristic lowers the utility of admission relative to opting out and is therefore associated with a greater tendency to choose the opt-out option; positive coefficients indicate a lower tendency to choose the opt-out option. ASC represents the baseline utility of the opt-out alternative and is not a DCE attribute. Random coefficients were assumed to follow normal distributions. “—” indicates that no random coefficient was estimated. **p < 0.01; ***p < 0.001.*

# Supplementary Table S4. Model fit statistics for latent class logit models

| Number of classes | Log-likelihood | Number of parameters | CAIC | BIC |
| --- | --- | --- | --- | --- |
| 2 | -2427.33 | 18 | 4977.53 | 4959.53 |
| 3 | -2347.61 | 28 | 4886.35 | 4858.35 |
| 4 | -2288.36 | 38 | 4836.11 | 4798.11 |
| 5* | -2209.47 | 48 | 4746.60 | 4698.60 |
| 6 | -2186.94 | 58 | 4769.79 | 4711.79 |
| 7 | -2174.97 | 68 | 4814.11 | 4746.11 |

Note: Lower values of information criteria indicate better model fit. The asterisk indicates the selected model.

# Supplementary Table S5. Level effects and class proportions from latent class regression

| Attribute | Level | Class 1 (21.7%) | Class 2 (16.4%) | Class 3 (20.6%) | Class 4 (28.5%) | Class 5 (12.8%) |
| --- | --- | --- | --- | --- | --- | --- |
| Clinical complexity | High (ref: low) | -4.761*** (-6.537, -2.986) | 0.036 (-0.219, 0.291) | -3.523*** (-5.196, -1.849) | -0.052 (-0.359, 0.255) | -1.003*** (-1.485, -0.521) |
| Length of stay vs. institutional benchmark | Above benchmark | 0.041 (-0.551, 0.632) | 0.383 (-0.012, 0.778) | -2.761** (-4.436, -1.087) | -0.165 (-0.516, 0.186) | -0.628** (-0.995, -0.260) |
| Treatment technology | Innovative | 0.246 (-0.321, 0.813) | 0.508*** (0.271, 0.746) | 6.113*** (3.335, 8.892) | 0.553** (0.226, 0.880) | 0.304* (0.036, 0.573) |
| Share of service revenue | Low (ref: high) | 0.643* (0.369, 1.249) | 0.249* (0.013, 0.484) | -7.894*** (-10.956, -4.833) | -0.782*** (-1.182, -0.382) | -0.141 (-0.481, 0.198) |
| Expected DIP margin per case | Break-even (ref: loss) | 3.881*** (2.196, 5.567) | 1.015*** (0.685, 1.345) | 7.276*** (4.071, 10.482) | 3.558*** (2.905, 4.211) | 1.308*** (0.876, 1.741) |
| Expected DIP margin per case | Profit (ref: loss) | 3.472*** (2.081, 4.863) | 0.812*** (0.377, 1.246) | 7.571*** (4.182, 10.960) | 4.385*** (3.601, 5.168) | 2.044*** (1.508, 2.580) |
| Compliance risk | Moderate (ref: none) | 0.556 (-0.285, 1.397) | 0.205 (-0.078, 0.489) | -0.213 (-1.617, 1.191) | -0.043 (-0.535, 0.448) | -0.896*** (-1.386, -0.407) |
| Compliance risk | High (ref: none) | 0.561 (-0.387, 1.508) | 0.363 (-0.064, 0.789) | 4.974** (2.009, 7.940) | -0.870** (-1.370, -0.369) | -1.535*** (-2.196, -0.873) |

Note: Data are expressed as β coefficients with 95% confidence intervals in parentheses. *p < 0.05; **p < 0.01; ***p < 0.001.
